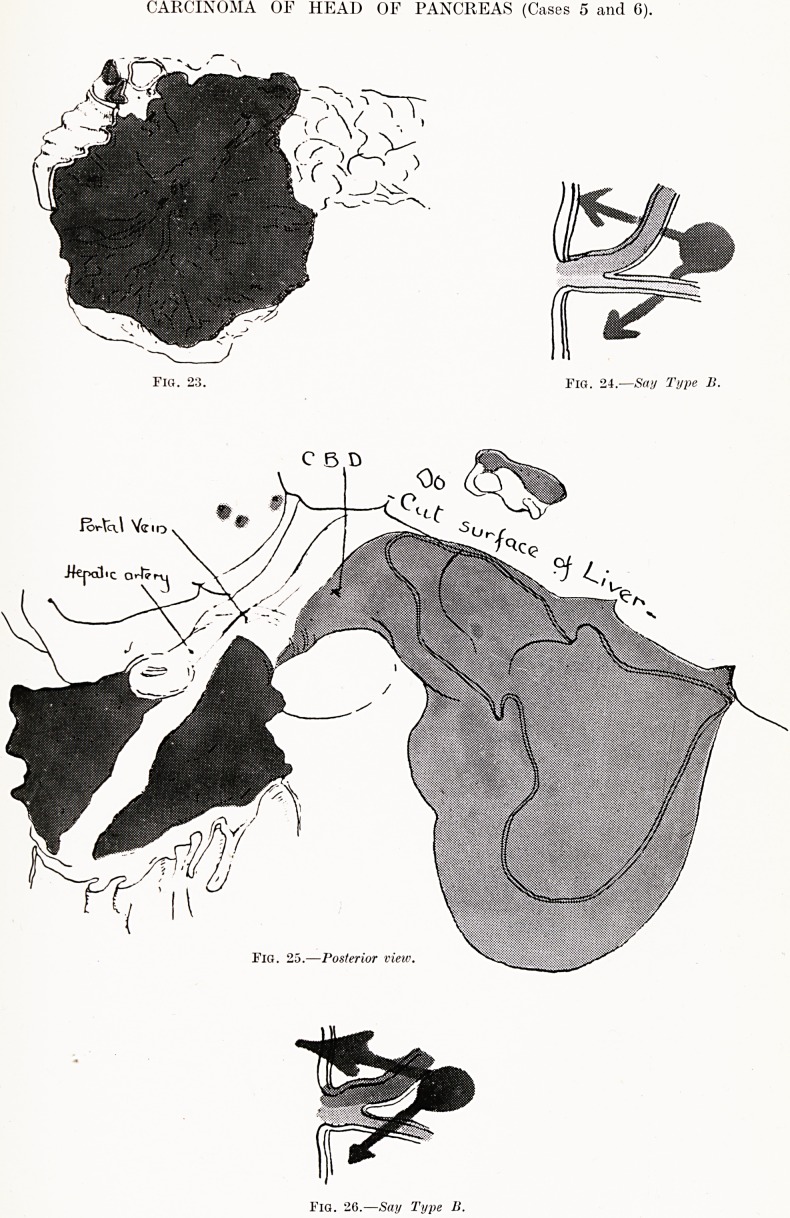# Six Cases of Cancer Arising in Tissues within the Duodenal Loop
1A Dissertation presented for a Martyn Memorial Scholarship, 1927.


**Published:** 1927

**Authors:** Helen B. Murgoci

**Affiliations:** Bristol General Hospital


### SIX CASES OF CANCER ARISING IN TISSUES WITHIN THE DUODENAL LOOP.[1]

BY

HELEN B. MURGOCI,

*Bristol General Hospital.*

---

Of the tumours found arising in the tissues surrounded by the loop of the duodenum carcinomata are the commonest. They rapidly produce symptoms due to obstruction of ducts. Their size is therefore small in proportion to the severity of the symptoms, which may produce a fatal result before any extensive infiltration or metastasis has taken place. The nature of the obstruction depends on the site of the growth, its direction and rate of extension, and the anatomical arrangement of the ducts.

*Site of Growth.*—The following epithelia, all normally included within an area of one square inch, may give rise to carcinoma. (Fig. 1.)

(1) Columnar epithelium of the duodenal papilla. Epithelium of Brunner's glands. Glandular epithelium of pancreatic rests in the duodenal wall.

(2) Columnar, plicated, gland-bearing epithelium of the ampulla of Vater.

[1] A Dissertation presented for a Martyn Memorial Scholarship, 1927.

(3) Columnar, gland-bearing epithelium of the lower end of the common bile-duct.

(4) Columnar epithelium of pancreatic ducts. Glandular epithelium of pancreatic acini. Irregular cubical cells of islets of Langerhans.

The site of origin of a cancer in this area is sometimes difficult to localise even on microscopical examination. Some clue is given by the histological character of the tumour, but this may not be of uniform type throughout. A classification, such as that given above, and based on the kinds of epithelia normally present, is generally adopted, but accurate localisation may be difficult when the tumour arises at a point where two types of epithelium merge into each other.

Statements of different observers as to the relative frequency of the various types of growth cannot be satisfactorily compared, for in addition to geographical and other variations, there are differences in the classifications used for obtaining the figures.

Pancreatic carcinoma is the commonest of those in this group. Hale White[1] found it in 5 per cent. of a series of cases of cancer ; 85 per cent. were in the head of the gland, Kaufmann[2] gave it as 1·76 per cent. of malignant tumours at autopsy ; Korte[3] as 2 per cent. of 2,943 malignant tumours examined.

Duodenal papilla carcinoma was found by Geiser[4] in 28 per cent. of cases of carcinoma, constituting 20 per cent. of duodenal carcinomata.

Cancer at the lower end of the common bile-duct was found in 23 of Rolleston's[5] 90 cases of cancer of the extra-hepatic bile-ducts. Colwell,[6] taking 3,300 cases of cancer found 0·33 per cent. in the extra-hepatic bile-duct. From these figures it appears that less than one in every thousand cases of cancer occurs at the lower end of the common bile-duct.

Cancer of the ampulla of Vater was found once in Colwell's[6] 3,300 cases. It is rare. Rolleston collected 19, Georges[5] 20, Outerbridge 100 cases.[5] The observers are not equally careful to differentiate these from the cancer of the duodenal papilla or bile-duct.

Juxta-ampullary cancer of Wirsung's duct is very rare. A few cases have been described. (Dr. Hadfield's paper gives three other references in the literature.)[8]

*Rate and Direction of Spread.*—Most of the cases end fatally in less than a year from the onset of symptoms. There are three main types of growth.

(1) Villous, papilliferous, rapidly growing and filling up the duct lumen. The cells are columnar. The growth may spread round the circumference of the duct. Its surface may ulcerate, as in the "cauliflower" growth at the duodenal papilla.

(2) Diffuse, accompanied by more fibrosis, extending along the walls of the duct. For example, the bile-ducts may be transformed into "cancer tubes." Of the same class is the scirrhosis cancer of the head of the pancreas. The cells are cubical and originate in glandular epithelium generally.

(3) Small localised nodules, which are hard from accompanying fibrosis. Bile-duct growths are commonly of this order.

Metastases are not produced by the papilliferous growths. They are most commonly found in cancer of the head of the pancreas. The diffuse and the localised growths may give rise to secondary growths in the adjoining lymph glands and in the liver.

Palpable tumours are according to Miraillé[7] produced by 25 per cent. of 113 cases of cancer of head of pancreas.

In the localities the primary growth does not produce a palpable tumour.

*The anatomical arrangement* of the ducts is an outcome of their development. The variations in development and in adult anatomy are illustrated by Figs. 2 to 14.*

Two factors which influence the origin of cancer are found in this area : (1) Development, (2) Irritation.

(1) The pancreas and the liver arise as buds from one part of the duodenum. Cell rests may be stranded and maintain their habit of growth.

(2) Irritation produces a reactive proliferation which may become uncontrolled. The irritation may be due to inflammation of glands or ducts, to gall-stones and pancreatic calculi, or to regurgitation of the contents of one duct into another. It may be due to excessive wear and tear in an area where normally three sets of ducts bring together three sets of fluids, each with very

**Possible Variations in Development.*

1. Common bile-duct may grow with dorsal pancreas, leaving Wirsung's duct alone. (Schirmer 4 times in 104 cases.) (Type G, Fig. 13.)
2. Ventral pancreas may develop from common stalk so that Wirsung's duct opens into common bile-duct some distance from the duodenum. (Type A, Fig. 7.)
3. " Separating septum " may be complete, giving rise to no ampulla, or may be less complete, giving rise to all degrees of length of ampulla or common biliary-pancreatic duct. (Mann.)
4. Ventral pancreas may grow out of wall of gut itself, near common duct, giving rise to separate openings, near each other. (Letulle and Nattan-Larrier.) (Type D, Fig. 10).
5. Pancreatic gland tissue may surround } The common bile-duct.
   ,, ,, ,, may not surround }
   (McConnell, *Jour. Anat. and Physiology*, XLIX.)
6. Santorini's duct may be main adult duct.
   ,, ,, ,, ,, almost independent, and patent. 4 of 100 cases.
   ,, ,, ,, not anastomose. 10 of 100 cases.
   ,, ,, ,, not be patent. 20 of 100 cases.
   } Numbers given by Opie, *Am. Med.*, Phila., 1903, vol. 5, p. 996.

different chemical and physical and physiological properties.

Although the only example of a rare type of growth, that of the juxta-ampullary part of Wirsung's duct, has already been reported by Dr. Hadfield, it has seemed worth while to bring this series together to illustrate the fact that clinical observation of the results of the different kinds of obstruction may furnish data as significant as those of experimental ligature. These results are briefly set out here in tabular form.

| Case. | Growths. | Obstruction of Bile-duct. | Obstruction of Pancreatic Duct | Obstruction of Duodenum. | Obstruction of Veins. | Pain. | Gall Bladder. |
|---|---|---|---|---|---|---|---|
| 1 | Malignant papilloma of duodenal papilla .. .. | + | + | + | — | Multiple. | Contracted. |
| 2 | Cancer of lower end of common bile-duct .. .. | + | — | — | — | + | ? |
| 3 | Cancer of lower end of Wirsung's duct .. .. .. | + | + | — | — | + | Dilated. |
| 4 | Cancer of head of pancreas .. .. | — | + | Slight. | + | + | Normal. |
| 5 | do. .. .. | + | + | Slight. | — | ? | ? |
| 6 | do. .. .. | + | + | + + | — | + | Dilated. |

All the cases here described occurred in males of ages between 48 and 68, and were fatal in six weeks to nine months after the onset of symptoms. In all cases but one there was jaundice, progressive and continuous. Operation was undertaken in two cases only. Both were unsuccessful, as the liver condition was diseased beyond recovery. All the pancreatic carcinomata had produced secondary growths. The juxta-ampullary carcinoma of Wirsung's duct had produced metastases in the adjoining glands.

**Case 1** (Figs. 15 and 16).—A. M., male, age 58. Was admitted to the General Hospital, Bristol, on October 15th, 1926, complaining of coughing up bile and of pain in epigastrium and left iliac fossa.

*History of Present Illness.*—Three weeks before admission he was ill and had a cough which was diagnosed as influenza. A week later he started to cough up bile, and had epigastric pain which was worse on coughing but was relieved after it. Jaundice appeared and the condition grew progressively worse.

*Past History.*—He had been admitted to the Hospital in 1915 on two occasions to have paracentesis abdominis performed. The pathological report on the ascitic fluid stated that no tubercle baccilli were found, and the cells were mainly lymphocytes.

*Condition on Admission.*—Very ill; jaundiced; unable to eat, sleep or breathe except in upright position. Fæces: clay coloured. Chest: Dullness found at right base. Rhonchi heard at both bases. Abdomen: tender, some distension, no tumours or enlarged viscera felt. Sputum: greenish-yellow, mucoid, about 8 ounces per day. No tubercle bacilli, streptococci or staphylococci were found. Blood: Wassermann reaction positive. X-ray: screen examination, showed a dull mass of a rounded shape in about the middle of the lower lobe of the right lung.

*Course of Illness.*—The signs in the chest became more evident. Wasting and jaundice increased. The cough for a time improved and less bile was brought up. There was no fever. He was continuously drowsy and wandering in mind. Operative treatment was not undertaken as the patient was too ill and the diagnosis obscure. Œdema of the legs and ascites appeared. The lung signs became extensive. Death occurred on January 9th, 1927.

*Post-mortem Examination.*—The transverse colon was found adherent to the anterior abdominal wall, so that the mesocolon

### PLATE VIII.

KEY OF TINTS.

Common bile-duct.

Duct of Wirsung.

Cancerous tissue.

FIG. 1.—*Possible origins of Cancer.* 1. *Duodenal papilla.* 2. *Ampulla of Vater.* 3. *Common bile-duct.* 4. *Duct of Wirsung.* 5. *Pancreas.* (After Rolleston—after Letulle.)

FIG. 2.—*Digestive tube from a* 2.5mm. (*fourth week*) *human embryo* (Peter Thompson).

FIG. 3.—*Fore gut from human embryo* (*fourth week*) (Broman).

FIG. 4.—*Fore gut from human embryo, 7 mm. long* (Peter Thompson).

FIG. 5.—(*Fifth week*).—*Growth of duodenum enables the dorsal and ventral pancreas to meet and their ducts to communicate.* (After Kollman.)

FIG. 6.—*Sixth week.*—11 *mm.*

NORMAL DEVELOPMENT OF DUCTS OF LIVER AND PANCREAS (FIGS. 2 to 6.)

(Figures from Gray's *Anatomy*, p. 140, 1918 ed., and *Human Embryology and Morphology* by Keith, pp. 249, 251, 264, 1912 ed.)

### PLATE IX.

### ANATOMY OF AMPULLA OF VATER.

Showing variations in termination of Common bile-duct (C.B.D.) and Wirsung's duct (D. of W.), and Duct of Santorini (D. of S.).

After (A—D only) Letulle and Nattan Larrier, *Bul. Soc. Anat. Paris*, 1899, p. 987.

(Type A.)

FIG. 7.—*No real papilla or ampulla. Wirsung's duct opens into common bile-duct.*

(Type B.)

FIG. 8.—*Papilla and ampulla.*

(Type C.)

FIG. 9.—*No papilla nor ampulla. Both ducts open into duodenum together.*

(Type D.)
FIG. 10.—*Ampullary dilatation of lower end of common bile-duct. Duodenal papilla. Two ducts open separately.*

(Type E.)

FIG. 11.—*No duct of Wirsung.*

(Type F.)
FIG. 12.—*Separate openings.*

(Type G.)

FIG. 13.—*Common bile-duct opens into duct of Santorini.*

(Type H.) (Similar to Bc C.)
FIG. 14.—*To show valve action of the tongue of mucous membrane between common bile-duct and duct of Wirsung in increased ampullary pressure.*
After D. Chamberlain.

Types C and D.—31% Mann, 28% D. Chamberlain on 100 cases. (*Brit. J. of Surgery*, Jan., 1927).

Type E.—2% D. Chamberlain, 4% Mann.

Type B.—35—33% Letulle and Nattan Larrier.

Type G.—4 in 104 Schirmer.

Type H.—Ampulla $\frac{1}{8}$ in., 23% D. Chamberlain, 45% Mann ; $\frac{1}{3}$–$\frac{1}{4}$ in., 47% D. Chamberlain, 20% Mann.

Type F.—20% Cunningham.

### PLATE X.

### CARCINOMA OF PAPILLA OF VATER (Case 1).

Fig. 16.—*Ampulla Type A. Columnar and round celled malignant papilloma.*

Fig. 15.

### CANCER OF LOWER END OF COMMON BILE-DUCT (Case 2).

Fig. 17.—*Posterior aspect.*

Fig. 18.—*Ampulla Type A*

### PLATE XI.

### CANCER OF JUXTA-AMPULLARY PART OF WIRSUNG'S DUCT (Case 3).

(Published by Dr. Hadfield, *Brit. Journ. of Surgery*, Vol. XII., No. 47, 1925.)

FIG. 19.

FIG. 20.—*Ampulla Type B approximating to D.*

### CANCER OF HEAD OF PANCREAS (Case 4).

FIG. 21.—*Posterior aspect.*

FIF. 22.—*Ampulla Type B (probably).*

### PLATE XII.

CARCINOMA OF HEAD OF PANCREAS (Cases 5 and 6).

Fig. 23.

Fig. 24.—*Say Type B.*

Fig. 25.—*Posterior view.*

Fig. 26.—*Say Type B.*

effectively cut off the perihepatic region from the lower one containing normal coils of small intestine. In the upper compartment a general suppurating mass full of adhesions was found, mainly around the gall-bladder. There was some ascitic fluid in the lower compartment.

The liver was small, puckered and its surface was nodular. The cut surface showed irregular but extensive interlobular cirrhosis. On the upper surface on the left side there was a large subphrenic pocket of pus. On the right side a necrotic mass, probably a broken-down gumma, was found, containing inspissated bile. This tracked through the diaphragm into a cavity in the lower lobe of the right lung, which communicated with a bronchus. This cavity contained inspissated bile and some small stones. It was about an inch across.

The gall-bladder wall was thickened and covered with adhesions. It was small. The common bile-duct was enormously dilated and full of small pigment stones and thick bile. On cutting it open, the lower end was found obstructed by a large very soft papilliferous growth at the duodenal papilla, which filled the lumen of the duodenum. The duct of Wirsung opened into this dilated bile-duct and was not markedly dilated. The substance of the pancreas appeared normal. The stomach was not dilated. It was involved in the suppurating perihepatic mass. The spleen was enlarged and hard as from chronic venous congestion. The lungs were both adherent to their pleuræ. Patches of broncho-pneumonia were found on both sides. There were some pneumonic areas on the right side. There was bronchitis and tracheitis. The heart was small and brown. Raised patches of atheroma were found in the aortic arch and abdominal aorta.

*Pathological Histology.*—Sections of the tumour showed an exuberant papilliferous growth. The cells were columnar and arranged on villus-like projections of the submucosa. The cell shape was not constant. In the deeper parts the cells became cubical, at the sides they were regular and cubical. In the centre of the fibrous tissue forming the stalk of the growth rods of cells were seen. These indicate that the growth was malignant and infiltrating the wall of the duodenum.

In the *liver* there was outstanding fibrosis at the periphery of lobules only. The hepatic veins were engaged and also the central hepatic sinusoids. The parenchyma was, in places, atrophied, most markedly at the centre of the lobules. Areas

Q

of necrosis were seen, and of fatty change at the centre and periphery. Small gummata with amorphous centres and fibrous envelope were found in some lobules near the periphery. The bile-ducts were not dilated. They were surrounded by a round cell infiltration. Their epithelium was denuded and in places showed catarrhal inflammation. Numerous proliferating bile-duct buds were seen in the perilobular fibrotic areas.

The *pancreas* showed a condition closely analogous to that seen in the liver. The fibrosis was extensive throughout the tissue, being most marked around the lobules, but also extending to isolate individual acini. The vessels were engorged and numerous. There was no endarteritis. The islets were well seen. The ducts were collapsed, but their lumen enlarged. The larger ducts and duct of Wirsung had somewhat fibrosed walls and irregular mucous membrane.

The pancreatic gland cells in some areas showed fatty change, in others they were more healthy. There were numerous small ducts which could often be seen leading into an acinus. The perilobular fibrous tissue showed the same appearance as that in the liver. It contained numerous rods of proliferating pancreatic duct buds.

A growth of relatively benign character, at the duodenal papilla, was fatal in thirteen weeks after onset of jaundice, by causing *obstruction* of the common bile-duct and pancreatic ducts. No metastases had taken place. Bile was still present in the gall-bladder. The clinical signs of obstruction of the common bile-duct and pancreatic duct were complicated and masked by the condition present in the liver and pancreas for years.

The lesions found post-mortem were found to be of two ages : (1) *Old* syphilitic cirrhosis of liver and pancreas, a well-marked cholecystitis and a probable perihepatitis dating from 1915. (2) *Recent* obstruction of bile and pancreatic ducts, producing dilatation and general jaundice. Recent softening of old gummata in the liver and establishment of a biliary-bronchial fistula; an upper abdominal mass of adhesions and some suppuration; a spreading broncho-pneumonia.

Microscopically the syphilitic lesions only are seen. The well-marked proliferation of bile and pancreatic duct buds, the localisation of the syphilitic lesion in liver and pancreas, the presence of stones in an inflamed gall-bladder, suggest that these may have been factors causing the development of neoplasm at the duodenal papilla.

There is no similar case recorded in the literature. A broken-down gumma producing a biliary-bronchial fistula is in itself rare. In no other reported case was this caused by an obstructing carcinoma.

**Case 2** (Figs. 17 and 18).—H. C. J., male, age 55. Was admitted to the General Hospital, Bristol, in December, 1926, complaining of aching in the back and jaundice.

*History of Present Condition.*—For six weeks before admission he had aching in the back. A week later jaundice appeared and progressively increased. There was continual loss of weight, a dull ache in the abdomen, and tenderness localised below and to the right of the umbilicus.

*Blood.*—Van den Bergh's reaction, direct and indirect, intensely positive, indicating an extra-hepatic obstruction and destruction of liver cells.

A diagnosis of pancreatic carcinoma was made and laparotomy performed. No growth was found in the pancreas. A cholecyst-gastrostomy was made to relieve the jaundice. Four days later he died, after having rigors and hematemeses.

*Post-mortem Examination.*—The head of the pancreas was somewhat hardened.

The common bile-duct was dilated. On opening it up a small growth was found at its lower end, just above the point of entrance of the pancreatic duct. The growth formed a localised thickening of the duct wall which had produced obstruction by obliterating the lumen. The growth was spreading along the walls of the common bile-duct, and had just reached the opening of the duct of Wirsung, causing it to become slightly dilated. There were no metastases.

Post-mortem changes in the tissues had taken place to such an extent that microscopical examination could not be made.

In spite of operation, the patient died six weeks after onset of symptoms. The growth was small, hard, and localised. The pancreatic duct was barely affected. No metastases had formed. There was abdominal pain. The condition could not have been diagnosed from any other obstruction of the common bile-duct.

**Case 3** (Figs. 19 and 20).—As this case has already been reported by Dr. Hadfield, a brief summary only is necessary here.

T. G., male, age 48. Was admitted to the General Hospital, complaining of jaundice and increasing weakness. These increased, and a diagnosis of cancer in the head of pancreas was made. An anastomosis between the gall-bladder and antero-superior surface of the stomach was performed, as the condition was thought to be due to non-calculous obstruction of the bile-duct in the head of the pancreas. The gall-bladder, on aspiration, contained thin, opalescent, practically colourless fluid. For three days the patient progressed satisfactorily, but collapse symptoms and some vomiting set in, and he died thirty-six hours after.

*Post-mortem Examination.*—Showed obstruction and dilatation of the common bile-duct and pancreatic duct by a small white spherical mass with roughened surface, arising at the posterior part of the termination of the pancreatic duct. The growth was strictly localised. It obstructed the common bile-duct by lateral pressure.

An accessory pancreatic duct was found opening separately into the duodenum. Four enlarged lymph glands were found behind the head of pancreas. The gall-bladder and intra-hepatic ducts were enlarged. The portal canals were prominent. Each lobule showed as a deep green spot fading to yellow-brown at its periphery. The surface of the liver was smooth, green and flabby. The pancreas tissue seemed normal.

*Pathological Histology.*—The growth showed a spheroidal-celled carcinoma in a dense fibrotic mass, and in an infected lymph gland the normal tissue was replaced by cells similar to those in the growth, except that here there was occasional tubule formation.

In the *liver* there was centrilobular pressure, atrophy and necrosis and bile stasis throughout the liver. The larger bile-ducts were most markedly dilated and their epithelium was desquamating ; all the ducts were surrounded by some inflammatory cell exudate. No other lesion was seen, other than that which might be associated with an uncomplicated obstruction to biliary flow.

The gland tissue of the pancreas was normal, except that around the lobules and ducts there was definite fibrosis and inflammatory cell reaction. The larger ducts were considerably dilated and their epithelium was flattened and hyperplastic in some places.

**Case 4** (Figs. 21 and 22).—W. W., male, age 61. Admitted to the General Hospital, Bristol, on October 21st, 1926, complaining of epigastric pain and loss of weight.

*History of Present Illness.* — For eight months had anorexia, loss of weight, constipation and pain in epigastrium, worse half to one hour after food. Some teeth were extracted and he felt better after.

*Condition on Admission.*—A tall man, previously strong, now feeling very weak. There was œdema of the feet, and a mitral systolic murmur. Abdominal examination revealed a hard mass in the epigastrium.

The urine yielded 1 per cent. of sugar. Test meal, barium meal, nothing abnormal. The fæces gave no evidence of pancreatic insufficiency, only delayed absorption of well-split fat.

*Course of Illness.* — The glycosuria disappeared after administering very little insulin and after dieting. The pulse had a small wave, and became irregular in force and frequency.

Pulsation was observed in the epigastrium. The liver edge was felt hard, rough and pulsating. Œdema appeared in the lumbar region and scrotum.

He died on November 15th.

*Post-mortem Examination.*—A carcinoma of the head of the pancreas was found, occluding the inferior vena cava slightly, and infiltrating the duodenal wall. The growth was not more than two inches across, and affected the head of the gland. It was hard. The common bile-duct opened into the duodenum at the summit of the papilla, which was above the level of the greater part of the tumour.

The liver had large secondary carcinomatous deposits under the peritoneum and also along the portal vessels inside the organ. These were hard, rounded, well localised nodules, surrounded by some hemorrhagic areas.

The gall-bladder was of normal size. It contained a few small stones. The heart showed evidence of old mitral disease. The right side was dilated. Both lungs showed basal broncho-pneumonic changes.

More secondary carcinomatous deposits were found in the peritoneum opposite those in the liver; also under the pleuræ and the pericardium.

Miraillé found that 82 cases of 113 of cancer of head of pancreas had jaundice. In this case the lesion was diagnosed, although the bile apparatus was not implicated.

**Case 5** (Figs. 23 and 24).—G. M., male, age 55. Admitted moribund to the Royal Infirmary, Bristol, in 1913.

*Post-mortem Examination.*—All tissues were deeply bile stained. The head of the pancreas was enlarged, nodular and hard. There was some central softening, and small hemorrhagic areas were found on section. The growth had reached the duodenal wall, and had involved the common bile-duct, causing it to dilate. There were generalised secondary growths throughout the liver, and the abdominal lymph glands were enlarged, especially the upper ones. Microscopical examination of secondary growths in the liver showed typical appearances of pancreatic cells.

As compared with the previous case, this one differs in that the growth, though of a similar nature, produced biliary obstruction but did not affect the portal vein.

**Case 6** (Figs. 25 and 26).—H. C., age 68. Admitted to the Royal Infirmary, Bristol, in 1900, complaining of jaundice and loss of weight. For some months he had felt ill. Seven weeks before admission jaundice, pain and vomiting had set in. On admission he was deeply jaundiced, very thin, and in pain. There was continual vomiting. A hard, globular, tender mass was felt under the right costal margin, possibly attached to the liver. Death took place in a few days.

*Post-mortem Examination.*—A fairly hard growth in the head of the pancreas was found, which had infiltrated the duodenal wall, occluded the common bile-duct, and become adherent to the transverse colon.

The stomach and pylorus were dilated, reaching almost to the symphysis pubis.

The liver had nodular secondary deposits all over its surface. The cut surface showed dilated bile-ducts and extensive bile-staining. The gall-bladder was dilated to two to three inches below the anterior border of the liver. It contained inky bile.

There were numerous adhesions of the ascending and descending colon to the abdominal wall. In some mesenteric glands were found secondary deposits. Adhesions were noted in the pleural cavities, with old calcareous deposits in the lung apices.

This case is similar to the previous one, but showed more extensive metastases and demonstrated dilatation of the gall-bladder. There was more involvement of the duodenum, producing occlusion and persistent vomiting and dilatation of stomach.

Grateful acknowledgment is due to Dr. Hadfield for permission to quote Case 3, to Dr. Symes for Cases 1 and 4, to Mr. Wood for Case 2, and to Dr. Fraser for Cases 5 and 6.

### REFERENCES.

[1] Hale White, *Clinical Journal*, 1900, vol. xvi., p. 17; *Guy's Hospital Report*, 1900, vol. liv., p. 17.

[2, 3] Ewing, *Neoplastic Diseases*, quotes Kaufmann and Korte.

[4] Geiser, German sources, quoted by Outerbridge, *Annals of Surgery*, 1913, vol. lvii., p. 407.

[5] Rolleston, *Diseases of Liver, Gall-Bladder and Bile-Ducts*, pp. 691, 703 in 1912 edition; Georges, *Thése*, Paris, 1896, quoted by Rolleston.

[6] Colwell, *Archives of Middlesex Hospital*, vol. v., 1905, p. 123.

[7] Miraillé, *Gazette des H pitaux*, 1893, quoted by Hillier and Goodall, *Archives of Middlesex Hospital*, vol. ii., p. 1.

[8] Hadfield, *British Journal of Surgery*, vol. xii., 1925, p. 47.

## Figures and Tables

**Fig. 1. Fig. 2. Fig. 3. Fig. 4. Fig. 5. Fig. 6. f1:**
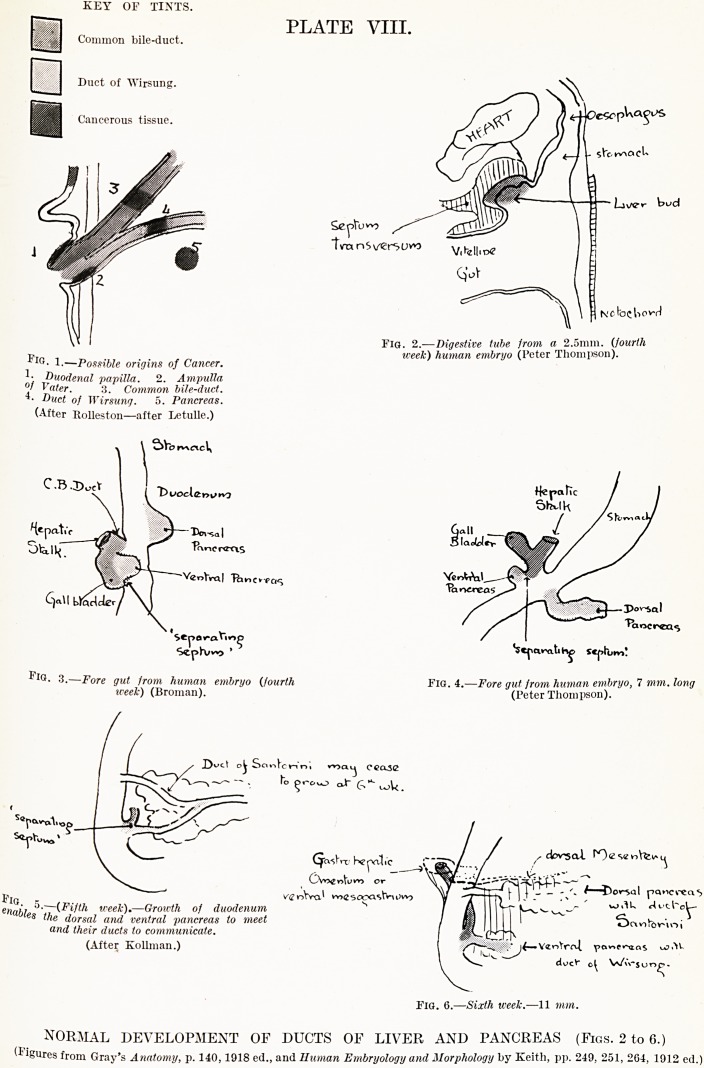


**Fig. 7. Fig. 8. Fig. 9. Fig. 10. Fig. 11. Fig. 12. Fig. 13. Fig. 14. f2:**
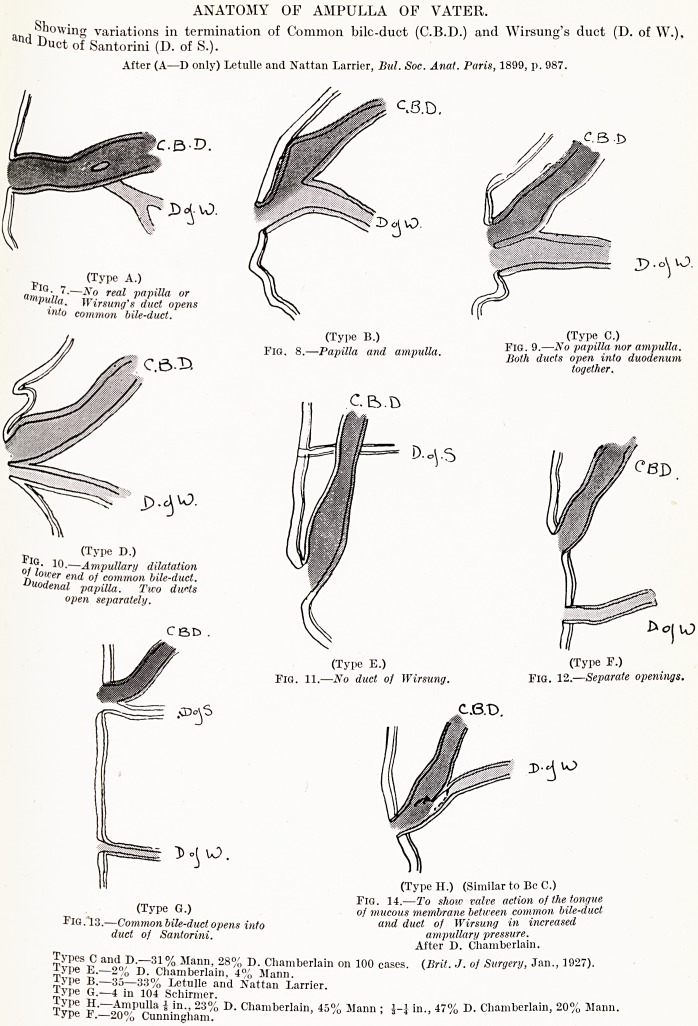


**Fig. 15. Fig. 16. f3:**
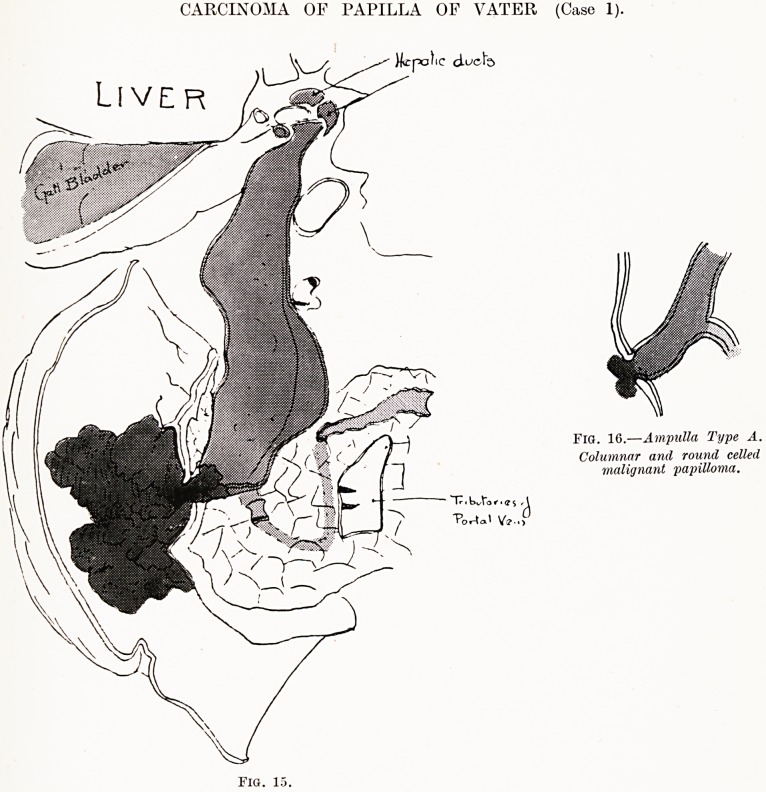


**Fig. 17. Fig. 18. f4:**
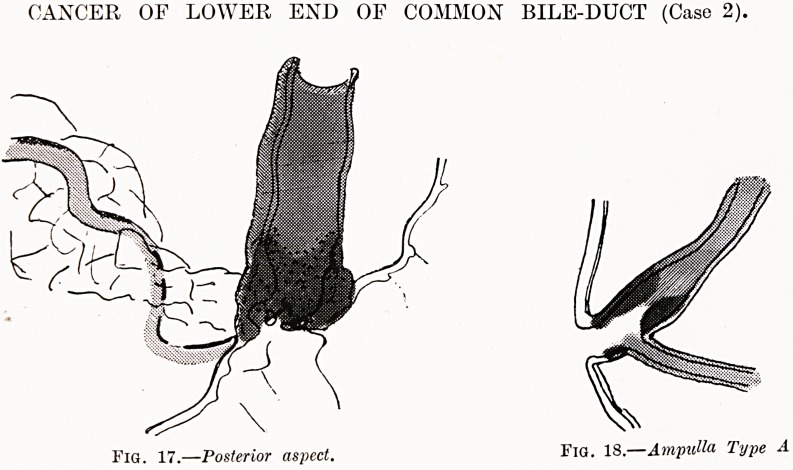


**Fig. 19. Fig. 20. f5:**
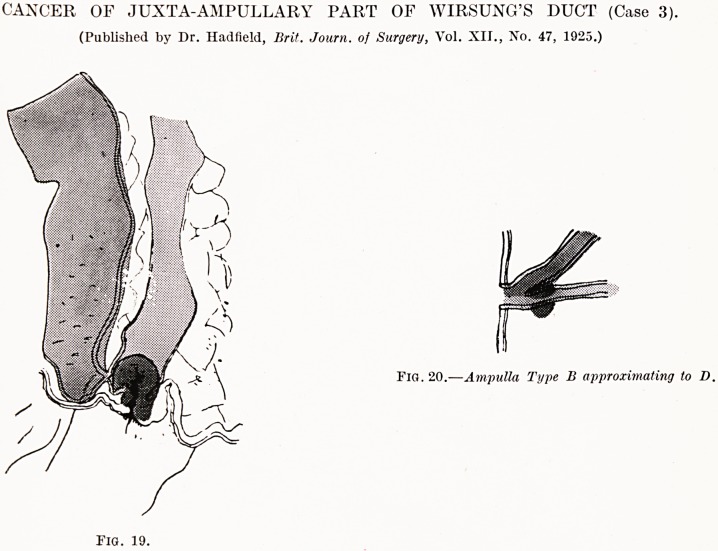


**Fig. 21. Fif. 22. f6:**
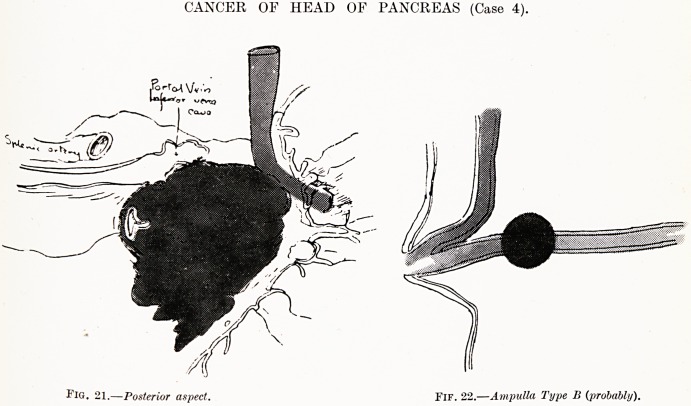


**Fig. 23. Fig. 24. Fig. 25. Fig. 26. f7:**